# Kinetic and structural analysis of human ALDH9A1

**DOI:** 10.1042/BSR20190558

**Published:** 2019-04-26

**Authors:** Radka Končitíková, Armelle Vigouroux, Martina Kopečná, Marek Šebela, Solange Moréra, David Kopečný

**Affiliations:** 1 Department of Protein Biochemistry and Proteomics, Centre of the Region Haná for Biotechnological and Agricultural Research, Faculty of Science, Palacký University, Šlechtitelů 27, CZ-783 71 Olomouc, Czech Republic; 2 Institute for Integrative Biology of the Cell (I2BC), CNRS-CEA-Univ. Paris-Sud, Université Paris-Saclay, Avenue de la Terrasse, F-91198, Gif-sur-Yvette, France

**Keywords:** 3-aminopropionaldehyde, 4-trimethylaminobutyraldehyde, aldehyde dehydrogenase, Homo sapiens, structure-function, X‐ray crystallography

## Abstract

Aldehyde dehydrogenases (ALDHs) constitute a superfamily of NAD(P)^+^-dependent enzymes, which detoxify aldehydes produced in various metabolic pathways to the corresponding carboxylic acids. Among the 19 human ALDHs, the cytosolic ALDH9A1 has so far never been fully enzymatically characterized and its structure is still unknown. Here, we report complete molecular and kinetic properties of human ALDH9A1 as well as three crystal forms at 2.3, 2.9, and 2.5 Å resolution. We show that ALDH9A1 exhibits wide substrate specificity to aminoaldehydes, aliphatic and aromatic aldehydes with a clear preference for *γ*-trimethylaminobutyraldehyde (TMABAL). The structure of ALDH9A1 reveals that the enzyme assembles as a tetramer. Each ALDH monomer displays a typical ALDHs fold composed of an oligomerization domain, a coenzyme domain, a catalytic domain, and an inter-domain linker highly conserved in amino-acid sequence and folding. Nonetheless, structural comparison reveals a position and a fold of the inter-domain linker of ALDH9A1 never observed in any other ALDH so far. This unique difference is not compatible with the presence of a bound substrate and a large conformational rearrangement of the linker up to 30 Å has to occur to allow the access of the substrate channel. Moreover, the αβE region consisting of an α-helix and a β-strand of the coenzyme domain at the dimer interface are disordered, likely due to the loss of interactions with the inter-domain linker, which leads to incomplete β-nicotinamide adenine dinucleotide (NAD^+^) binding pocket.

## Introduction

Aldehyde dehydrogenases (ALDHs) catalyze the NAD(P)^+^-dependent irreversible oxidation of aliphatic and aromatic aldehydes. To date, 24 *ALDH* gene families have been identified in genomes of eukaryotic species and 19 *ALDH* genes in *Homo sapiens* [1,2]. Human ALDH9A1 is a cytosolic tetrameric enzyme of ∼216 kDa that was first purified from the liver and characterized as a 4-aminobutyraldehyde (ABAL) dehydrogenase (ABALDH, E.C. 1.2.1.19) [3]. In mammalian organisms, ABAL is formed via an oxidative deamination of the biogenic amine putrescine by diamine oxidase (E.C. 1.4.3.22) [4]. Its oxidation by ALDH9A1 results in the formation of γ-aminobutyric acid (GABA), which is a well-known neurotransmitter.

*ALDH9A1* gene is highly expressed in the liver, skeletal muscle, kidney, pancreas, and heart [5]. The enzyme is also present in the brain and the spinal cord [6]. The enzyme displays activity with a dopamine metabolite 3,4-dihydroxyphenylacetaldehyde (DOPAL) [7] and much higher with betaine aldehyde (BAL) [6,8] leading to the production of glycine betaine (therefore often annotated as BADH, E.C. 1.2.1.8). Glycine betaine is a quaternary ammonium compound acting as a zwitter-ion at physiological pH and maintaining protein and membrane conformations under various stress conditions [9].

**Figure 1:**
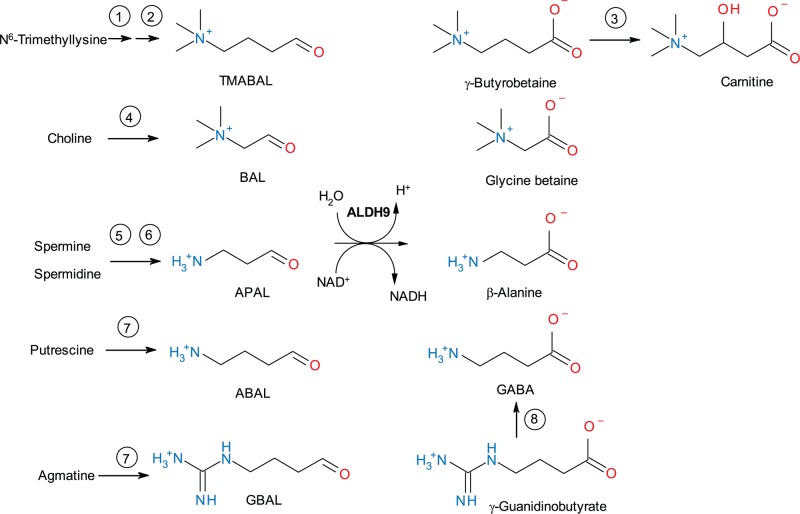
A scheme of possible reactions catalyzed by human ALDH9A1 The enzyme can catalyze conversion of TMABAL, BAL, and aminoaldehydes such as APAL, ABAL, and GBAL. The involved enzymes are: 1 – *N*^6^-trimethyllysine hydroxylase; 2 – 3-hydroxy-*N*^6^-trimethyllysine aldolase; 3 – γ-butyrobetaine dioxygenase; 4 – choline dehydrogenase; 5 – spermine oxidase; 6 – polyamine oxidase; 7 – diamine oxidase; 8 – agmatinase. Abbreviations: APAL, 3-aminopropionaldehyde; GBAL, 4-guanidinobutyraldehyde.

A mammalian ALDH displaying *γ*-trimethylaminobutyraldehyde (TMABAL) dehydrogenase (TMABALDH, E.C. 1.2.1.47) activity was first purified from bovine liver [10]. Later on, other mammalian isoforms from rat and human were shown to be TMABALDHs thus it became clear that TMABALDH and ALDH9A1 were indeed the same enzyme [11]. The enzyme is involved in the carnitine synthesis pathway comprising three other enzymes: trimethyl lysine dioxygenase, 3-hydroxy-*N*-trimethyllysine aldolase and γ-butyrobetaine (TMABA) dioxygenase (Figure 1). Carnitine (3-hydroxy-4-*N*-trimethylaminobutyrate) is a water-soluble quaternary amine, which transports the CoA-activated fatty acids into the mitochondrial matrix for β-oxidation as well as products of peroxisomal β-oxidation, for their full oxidation to CO_2_ and H_2_O in the Krebs cycle [12]. Both, carnitine synthesis and uptake are regulated by the peroxisome proliferator-activated receptor α (PPARα), which is a transcription factor involved in lipid metabolism and energy homeostasis. It is abundantly expressed in tissues showing high rates of β-oxidation such as liver and kidney [13]. It has been shown that PPARα regulates the expression of *ALDH9A1* gene [14].

ALDH9A1 may play a role in systemic vasculitis and vasculitis-associated diseases such as Kawasaki disease known as a mucocutaneous lymph node syndrome [15]. Patients’ serum with the Kawasaki disease contains significantly induced levels of anti-ALDH9A1 antibodies. Decreased expression of *ALDH9A1* may also contribute to a non-alcoholic steatohepatitis without inflammation as reported by the study on a mice model [16]. Allelic variants in *ALDH9A1* gene have also been observed [5]. Polymorphism in several genes related to GABA signaling pathway including *ALDH9A1* has been associated with neuroleptic-induced tardive dyskinesia, which is an involuntary movement disorder that develops in patients undergoing a long-term treatment with antipsychotic medications [17].

Herein, we report a complete enzymatic characterization and the structure of human ALDH9A1 in three different crystal forms. Kinetic parameters and substrate specificity were determined using various aminoaldehydes including 3-aminopropanal (APAL) or 4-guanidinobutyraldehyde (GBAL), which have so far never been analyzed with this enzyme. ALDH9A1 is one of the few remaining members of the human ALDH superfamily with yet unknown crystal structure. Although the enzyme was co-crystallized with the β-nicotinamide adenine dinucleotide (NAD^+^) coenzyme, all structures, which are very similar, are devoid of NAD^+^ and display the same disordered region forming the coenzyme binding site. Structural comparison with the cod liver ALDH9A2 (PDB ID: 1BPW) and other ALDHs revealed that the inter-domain linker of human ALDH9A1, involved in the coenzyme binding, adopts a position and a fold that have never been observed so far in any X-ray structure of ALDHs.

## Results

### Substrate specificity and kinetic parameters

**Figure 2:**
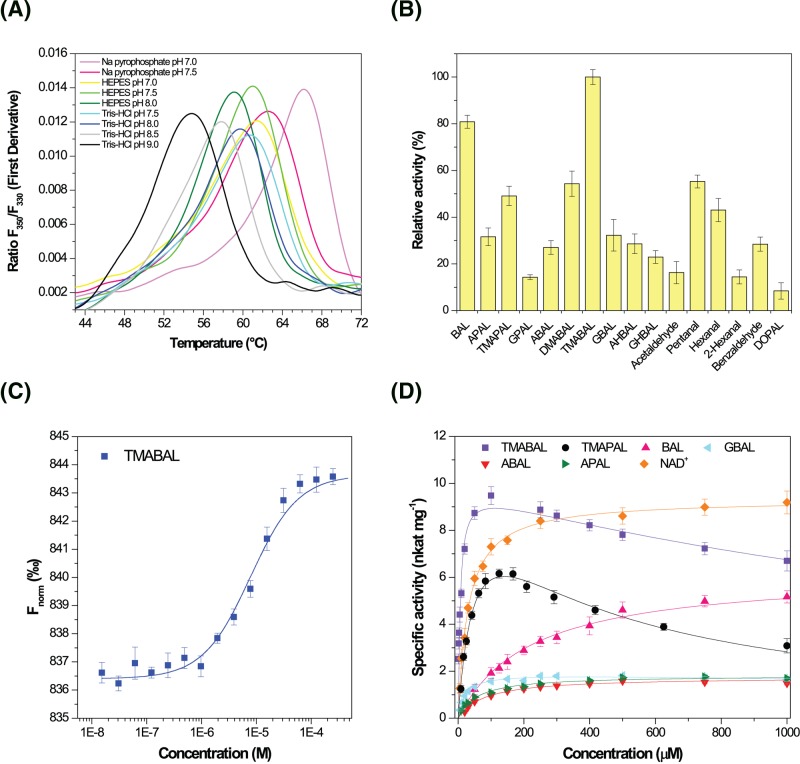
Kinetic and molecular properties of human ALDH9A1 (**A**) **Effect of pH on the thermal unfolding of HsALDH9A1 measured by nanoDSF**. All buffers were at 150 mM concentration. (**B**) **Screening of substrate specificity**. Measurements were performed with 1 mM substrate in 150 mM sodium pyrophosphate, pH 7.5 containing 1.0 mM NAD^+^. Specific activity values with TMABAL were arbitrarily taken as 100%. Error bars stand for S.D. from four measurements. (**C**) **MST binding curve for TMABAL**. The enzyme was fluorescently labeled using RED-Tris-NTA dye for the binding measurement with substrate and coenzyme on a Monolith NT.115 instrument. F_norm_ stands for a normalized fluorescence. (**D**) **Saturation curves for putative *in vivo* substrates**. The data were measured with 1.0 mM NAD^+^. Those for NAD^+^ were measured using 150 µM TMABAL in 150 mM sodium pyrophosphate, pH 7.5. Abbreviation: MST, microscale thermophoresis; AHBAL, 4-amino-2-hydroxybutyraldehyde; GHBAL, 4-guanidino-2-hydroxybutyraldehyde.

The human *ALDH9A1* (GenBank ID: AF172093) is a protein of 494 amino acids (Uniprot ID: P49189). A new polymorphism site was discovered in cDNA used in this work at the position 330. It appears in the codon for Ile^110^ (ATT is changed to ATC). However, this variation does not alter the amino acid composition. Kinetic measurements were performed at the known optimal pH of 7.5, as described in previous studies [3,8,11]. The enzyme is very sensitive and becomes nearly inactive after two re-freezing cycles. The effect of the buffer composition on protein stability was further investigated using nano-differential scanning fluorimetry (nanoDSF) (Figure 2A) and the highest melting temperatures were observed in buffers at pH 7.0 and 7.5.

Several aminoaldehydes were screened as potential substrates of ALDH9A1 using a fixed 1 mM concentration of the NAD^+^ coenzyme. The enzyme displays a wide range of substrate specificity (Figure 2B). Indeed, although ALDH9A1 shows the highest rate activity for TMABAL and BAL, it can oxidize other aminoaldehydes such APAL, ABAL, *γ*-dimethylaminobutyraldehyde (DMABAL) or GBAL. In line with previous studies where specific activities of 14, 1.8, and 2.3 nmol.s^−1^.mg^−1^ using ABAL were reported for the enzyme isolated from liver and brain [3,6,8], we measured a final activity of ∼1.8 nmol.s^−1^.mg^−1^. The enzyme can also convert aliphatic aldehydes such as acetaldehyde, hexanal, or 2-hexanal (lipid peroxidation product) as well as aromatic aldehydes such as benzaldehyde and DOPAL. The *K*_m_ value for NAD^+^ of 32 ± 2 μM correlates well with the measured *K*_d_ value of 16 ± 3 μM and the previous reported *K*_m_ value of 13 µM [3]. As the activity with NADP^+^ is only ∼2–3% of that with NAD^+^, NAD^+^ is the preferred coenzyme for HsALDH9A1.

Kinetic properties of ALDH9A1 were further explored (Table 1). A comparison of the catalytic efficiency values (*V*_max_/*K*_m_) shows that TMABAL is the best substrate *in vitro*. The enzyme displays a *K*_m_ value of 6 ± 1 μM (*V*_max_ ∼ 9.8 nmol.s^−1^.mg^−1^) for this substrate while that for BAL is much higher 216 ± 16 μM (*V*_max_ ∼ 6.3 nmol.s^−1^.mg^−1^). Dissociation constants (*K*_d_) of 6 ± 3 μM for TMABAL (Figure 2C) and 171 ± 35 μM for BAL were measured by microscale thermophoresis (MST) in the absence of the coenzyme. These values are close to the respective *K*_m_ values. Together with the catalytic efficiency values, they indicate that ALDH9A1 should be first of all a TMABALDH *in vivo*. Nonetheless, the putative *in vivo* substrates ABAL, APAL, and GBAL which share similar saturation curves can be oxidized with a *V*_max_ value approximately five-fold lower than those for TMABAL and *K*_m_ values in low micromolar range (Figure 2D and Table 1). Indeed, a *K*_m_ value of 13 µM for ABAL with a *V*_max_ value of 33 nmol.s^−1^.mg^−1^ was previously reported for the native enzyme [3] as well as *K*_m_ values of 5 and 260 µM for TMABAL [11] and BAL [8], respectively.

**Table 1:** Kinetic and affinity parameters for human ALDH9A1 and selected substrates

Ligand	*K*_m_ (µM)	*V*_max_ (nmol.s^−1^.mg^−1^)	*V*_max_*/K*_m_ (relative)	*K*_i_ (µM)	*K*_d_ (µM)
NAD^+^	32 ± 2	*9.3 ± 0.2	-	-	16 ± 3
TMABAL	6 ± 1	9.8 ± 0.4	1	2200 ± 590	6 ± 3
DMABAL	21 ± 4	4.7 ± 0.3	0.13	10880 ± 1350	n.d.
ABAL	67 ± 7	1.8 ± 0.1	0.02	-	n.d.
GBAL	21 ± 2	1.9 ± 0.1	0.05	7230 ± 1650	n.d.
TMAPAL	53 ± 6	9.9 ± 0.1	0.11	470 ± 45	n.d.
APAL	56 ± 7	1.8 ± 0.1	0.02	-	n.d.
BAL	216 ± 16	6.3 ± 0.2	0.02	-	171 ± 35
Acetaldehyde	17 ± 3	0.8 ± 0.1	0.03	-	n.d.
Valeraldehyde	35 ± 5	3.6 ± 0.1	0.06	-	n.d.
Hexanal	50 ± 16	2.9 ± 0.1	0.03	-	n.d.
2-Hexenal	26 ± 2	1.0 ± 0.1	0.02	-	n.d.
DOPAL	11 ± 1	0.5 ± 0.1	0.03	-	n.d.

*V*_max_/*K*_m_ ratios are expressed in relative values referring to the best TMABAL substrate (*V*_max_/*K*_m_ = 1). Saturation curves for aldehydes were measured in 150 mM sodium pyrophosphate, pH 7.5, using 1 mM NAD^+^; saturation curve for NAD^+^ was measured using 150 µM TMABAL. Kinetic constants including their standard error values were determined using GraphPad Prism 5.0 software including *K*_i_, which is the substrate inhibition constant. The lower *V*_max_ value for NAD^+^ (indicated by asterisks) compared with that for TMABAL results from using a fixed sub-saturating TMABAL concentration in the saturation of the enzyme by NAD^+^. *K*_d_ values were determined using MST. Abbreviations: n.d., not determined; TMAPAL, *N,N,N*-trimethyl-3-aminopropionaldehyde.

### Crystal structure of HsALDH9A1

**Figure 3:**
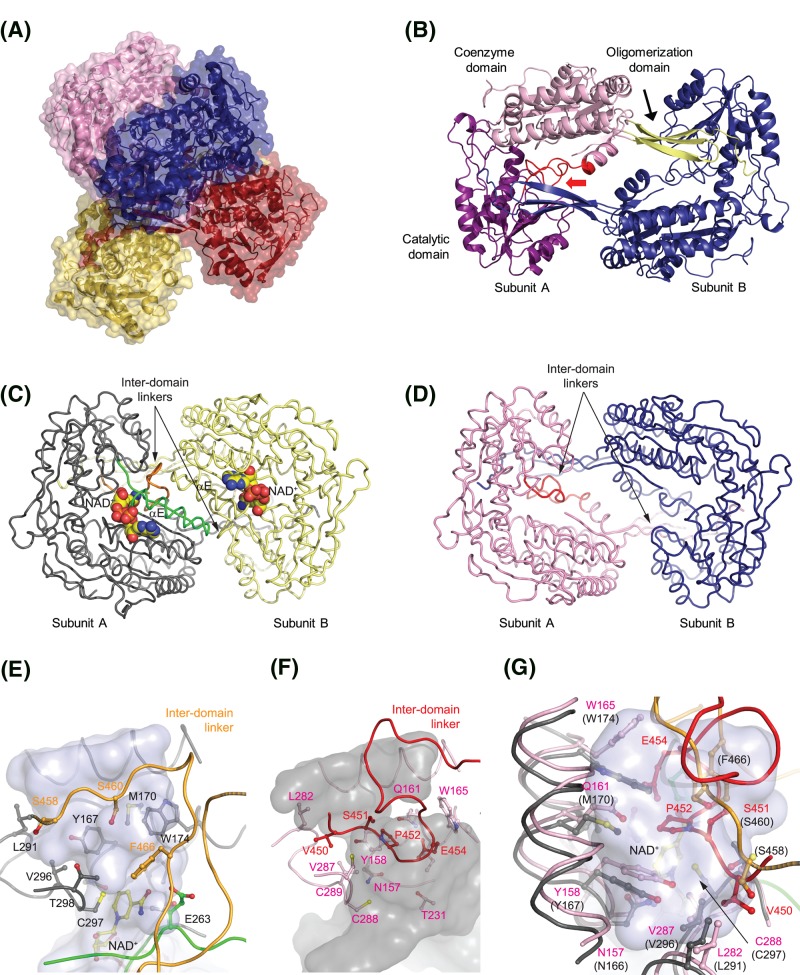
Crystal structure of human ALDH9A1 (**A**) **The classical ALDH tetramer of ALDH9A1**. Each subunit has single color and is shown in surface representation. (**B**) **The domain-swapped dimer**. One subunit is colored according to three domains and highlights a domain architecture while the other subunit has a single blue color. The inter-domain linker is colored red. (**C**) **The dimer of GmALDH9A2**. The dimer (PDB ID: 1BPW) shown as a gray- and yellow-colored ribbon for subunits (A,B). The αE-helix and βE-strand at the dimer interface of GmALDH9A2 (subunit A) are colored in green, while the inter-domain linker is in orange. The NAD^+^ molecules are in atom-coded colors. (**D**) **The dimer of HsALDH9A1.** The dimer (PDB ID: 6QAK, this work) is shown as a pink- and blue-colored ribbon for subunits (A,B). The αβE section is absent and the inter-domain linker is in red. (**E**) **A side-view at the substrate channel and the inter-domain linker in GmALDH9A2.** GmALDH9A2 is shown in gray and labeled in black while its inter-domain linker is in orange. (**F**) **A side-view at the substrate channel and altered fold of the inter-domain linker in HsALDH9A1.** HsALDH9A1 residues and ribbons are shown in pink and pink labelled while the inter-domain linker is in red (red labeled). (**G**) **A top-view of superposed substrate channels.** Ribbon, residue and label colors follow those in panel (E) and (F).

Crystallization of the HsALDH9A1 apoform was not successful. Co-crystallization with 50 mM NAD^+^ with or without 10 mM TMABA product resulted into different crystal forms. However, the three solved structures from P2_1_2_1_2, P2_1_, and C2 space groups at 2.5, 2.9, and 2.3 Å, respectively (Table 2), reveal an ALDH9A1 apoform in the absence of a bound NAD^+^ or TMABA. The structures were determined by molecular replacement using the structure of BADH from cod (*Gadus morhua* subsp. *callarias*) liver as a search model (PDB IDs: 1BPW and 1A4S; 71% sequence identity) [19]. This enzyme is also annotated GmALDH9A2 [1]. The asymmetric units of the P2_1_2_1_2 and P2_1_ structures contain two similar tetramers (dimer-of-dimers) and that of C2 only one (Figure 3A). The tetrameric form in solution with a molecular mass value of 214 ± 12 kDa was also confirmed by gel permeation chromatography (monomer of 55.4 kDa including the His-tag). All monomers are very similar to each other with an RMSD up to 0.24 Å. Superposition with monomers of GmALDH9A2 gives an RMSD of 1.3–1.6 Å. Each monomer displays the classical ALDH fold consisting of a catalytic domain (residues 258–448) with the catalytic Cys^288^, a coenzyme binding domain (residues 1–127, 146–257, 470–478), and an oligomerization domain (residues 128–145 and 479–494), which wraps over the groove between the catalytic and coenzyme domains of the other monomer forming the dimer (Figure 3B).

**Table 2:** Data collection and refinement statistics

	HsALDH9A1		
PDB ID	6QAK	6QAO	6QAP
Space group	P2_1_2_1_2	P2_1_	C2
Asymmetric unit	2 tetramers	2 tetramers	1 tetramer
Unit cell (Å)			
a	160.4	113.7	164.3
b	159.6	167.4	160.0
c	160.6	116.1	84.6
α = γ (°)	90.0	90.0	90.0
β (°)	90.0	90.9	91.1
Resolution (Å)	48.4-2.50 (2.65-2.50)	49.7-2.9 (3.07-2.9)	47.7-2.3 (2.4-2.3)
Observed reflections^1^	1917659 (297022)	686717 (107954)	682716 (104713)
Unique reflections	142787 (22393)	96373 (15121)	96851 (15363)
Completeness (%)	99.6 (97.8)	99.5 (97.1)	99.7 (98.1)
*I/σ (I)*	10.3 (1.0)	7.3 (1.2)	9.9 (1.4)
*R*_sym_	0.200 (2.586)	19.1 (1.3)	14.1 (1.2)
*R*_meas_	0.208 (2.689)	20.7 (1.5)	15.2 (1.3)
CC_1/2_^2^	99.8 (50.5)	99.5 (55.4)	99.8 (58.9)
*R*_cryst_ (%)	19.0	17.8	22.0
*R*_free_ (%)^3^	22.0	21.3	25.2
RMSD bond lengths (Å)	0.01	0.01	0.01
RMSD bond angles (°)	1.17	1.18	1.16
Mean B value (Å^2^):			
chain A/B/C/D/	75/73/83/83	82/89/101/78	62/67/63/62
E/F/G/H	91/82/72/87	92/93/89/93	
Solvent	53.2	65.0	52.8
Clash score^4^	4.1	4.21	4.42
MolProbity score^4^	1.86	2.09	1.84
Ramachandran statistics (%)^4^			
Favored	98.18	97.15	97.48
Outliers	0.08	0.4	0.05

1 Numbers in parentheses represent values in the highest resolution shell.

2 *CC_1/2_*stands for a percentage of correlation between intensities from a random half-dataset.

3 The 5% test set.

4 Generated with MolProbity [18].

The first remarkable difference concerns the region comprising residues 232–256 of HsADLH9A1 which belongs to the coenzyme domain and usually forms the dimer interface in other known ALDHs. The region αβE, named according to the nomenclature for GmALDH9A2 [19], is composed of the αE helix followed by the βE strand. The αβE region is not visible in the electron density maps thus is highly mobile (Figure 3C,D). The αE helix delineates the coenzyme cavity and possesses conserved residues known to bind the pyrophosphate moiety such as Ser^233^ and Thr^236^ (Ser^242^ and Thr^245^ in GmALDH9A2). The βE strand defined by residues 250–254 would be a part of the central five-stranded pleated β-sheet of the coenzyme domain (abbreviated as Rossmann fold). The last residue of this strand is indeed the conserved active-site base glutamate Glu^254^. The remaining residues 255–257 bridge the gap over the nicotinamide riboside moiety and connect to the catalytic domain.

### Inter-domain linker and active site

The inter-domain linker (residues 449–470), which does not adopt the typical fold so far observed in all known ALDHs’ structures (Figure 3E,F), represents the second major difference in HsALDH9A1. Under classical fold, this highly conserved region has an important role both in stabilizing the coenzyme binding site and in interacting with the bound substrate. Indeed, it protrudes alongside the edge between the coenzyme and the catalytic domains and establishes several H-bonds including the βE strand forming the coenzyme binding site. In the HsALDH9A1 structure, this inter-domain region adopts a new position and fold, associated with large conformational changes compared with GmALDH9A2 structure [19]. This is not compatible with a bound substrate (Figure 3G). For example, Lys^461^-Lys^462^-Ser^463^-Gly^464^ are located between 23 and 30 Å away from their equivalent residues in GmALDH9A2 and Pro^452^-Val^453^ and Glu^454^ (10 Å away from their equivalent residues in GmALDH9A2) occupy the substrate binding site.

**Figure 4:**
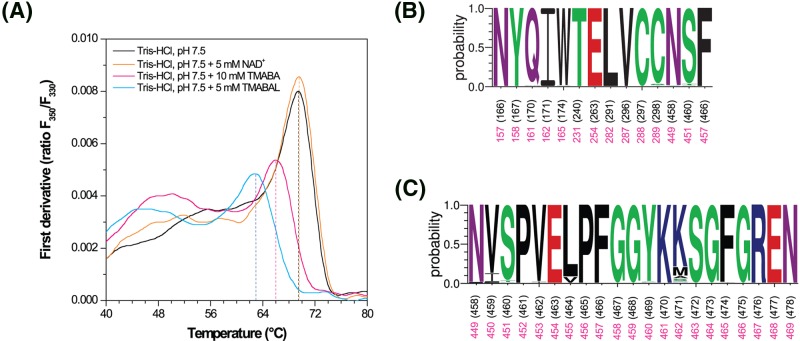
Unfolding of ALDH9A1 and conservation of the active site in ALDH9 family (**A**) **Effect of the coenzyme presence on the thermal unfolding of human ALDH9A1**. Measured by nanoDSF on Tycho NT.6 instrument using 150 mM Tris/HCl, pH 7.5 buffer and 5 mM substrate TMABAL, 5 mM coenzyme NAD^+^ and 10 mM product TMABA. (**B**,**C**) **Conservation of amino acid residues forming the active site (B) and the inter-domain linker (C) in members of the ALDH9 family**. Chosen residues and numbering follows that of GmALDH9A2 (labeled in black). Corresponding residues in HsALDH9A1 are labeled in pink. Sequence logo was made using WebLogo 3 (http://weblogo.threeplusone.com).

NanoDSF experiments with the purified apo-enzyme and enzyme with either 5 mM NAD^+^, or 5 mM substrate, or 10 mM product were performed to check transitions in the folding state of HsALDH9A1. While a high single peak was present for the ALDH9A1 apoform as well as for the coenzyme complex (69.5°C, Figure 4A), the enzyme became slightly destabilized with the substrate TMABAL (63°C) and the product TMABA (66°C). Moreover, a broad transition was observed at lower temperatures between 45 and 52°C for both the substrate and product complexes indicating possible conformational changes compared with the apoform.

## Discussion

In the present study, using kinetic assays and affinity measurements, we showed that among various available aminoaldehydes, ALDH9A1 preferentially oxidizes TMABAL with the highest rates at very low saturating micromolar concentrations. This suggests that the major *in vivo* role of this enzyme is a TMABALDH activity resulting to TMABA and further carnitine production, especially in liver. However, in other organs, the enzyme may be involved in oxidation of other aminoaldehydes and aliphatic aldehydes. While the involvement of ALDH9A1 in the production of the GABA neurotransmitter from ABAL was discussed in the past [6], its role in degradation of APAL or GBAL has not been considered at all. These aldehydes were previously deeply studied for plant ALDH10 family, the closest relative of the ALDH9 family [20,21].

APAL is known to cause apoptotic and necrotic death of both neurons and glial cells during cerebral ischemia [22]. As *ALDH9A1* is expressed in the brain, it is very likely that it oxidizes ABAL as well as APAL in this organ. It is well known that APAL is produced by polyamine oxidase mediating the oxidation of spermine and spermidine [23] and by spermine oxidase releasing spermidine and hydrogen peroxide [24]. Moreover, APAL can be non-enzymatically converted into acrolein, which is even more toxic than hydrogen peroxide [25]. On the other hand, GBAL oxidation represents another way of GABA production (predominantly, it is generated by the cytosolic glutamate decarboxylase, E.C. 4.1.1.15). GBAL is produced from agmatine as demonstrated with swine kidney diamine oxidase [26]. Kidneys may be a place for further GBAL conversion into γ-guanidinobutyrate, which is likely hydrolyzed to GABA by agmatinase. Again, this enzyme is highly abundant in the liver and kidney [27]. Oxidation of BAL to glycine betaine apparently requires higher BAL concentrations, which is different from the other aminoaldehydes. The *K*_m_ value of 182 µM for ALDH9A1 was reported for BAL with catalytic efficiency similar to ABAL and only 3% of that for TMABAL [11]. Glycine betaine is known to contribute to a normal homocysteine metabolism by donating its methyl group for the remethylation of homocysteine to methionine. Again, the major site of betaine metabolism is liver [28,29].

The structural comparison with the cod liver ALDH9 (PDB 1BPW) revealed two major differences likely linked to each other. The first one concerns the αβE region, which is composed of the α-helix E and β-strand E, located at the dimer interface. This region interacts with the bound NAD^+^ when present in ALDH structures, and the absence of a bound NAD^+^ in the HsALDH9A1 structure is in agreement with this largely disordered region. Moreover, with or without a bound coenzyme, this region has always been observed in X-ray structures in contact with the inter-domain linker located in the C-terminal part of the enzyme. The active site residues as well as those forming the inter-domain linker are highly conserved among ALDH9 family members (Figure 4B,C). Only two residues are different in the active site of GmALDH9A2 compared with HsALDH9A1. First of all, the catalytic cysteine, which is nearly always followed by the second cysteine at the neighboring position (Cys^289^ in HsALDH9A1), is the threonine residue (Thr^298^) in the cod enzyme. Second, the highly conserved glutamine Gln^161^ in ALDH9A1 is substituted by the methionine Met^170^ in GmALDH9A2. The cod enzyme, which has only been studied with six substrates [30], displays the highest catalytic efficiency for BAL followed by benzaldehyde. As no other aminoaldehydes including TMABAL were tested, it is difficult to deduce any effect of these two substitutions on substrate specificity. *K*_m_ value of 140 µM and *V*_max_ of 15 nmol.s^−1^.mg^−1^ for BAL are comparable with those presented in the present study for HsALDH9A1 (216 µM and 6.3 nmol.s^−1^.mg^−1^).

**Figure 5:**
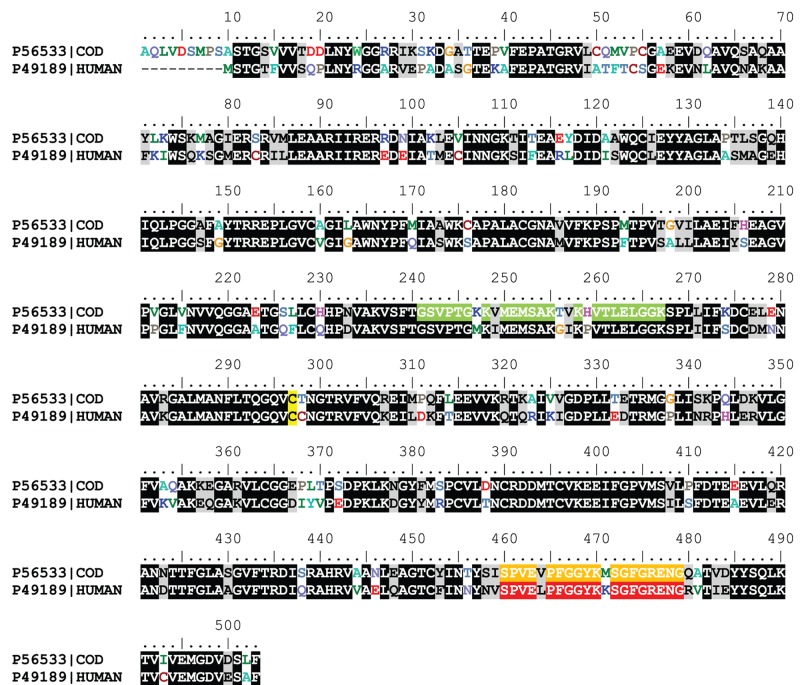
Sequence alignment of cod liver ALDH9A2 (Uniprot ID: P56533) and HsALDH9A1 (Uniprot ID: P49189) Catalytic cysteine is shown in yellow. The αE-helix and βE-strand at the dimer interface of GmALDH9A2 (and disordered in HsALDH9A1) are colored in green. The inter-domain linker is colored in orange for GmALDH9A2 and red for HsALDH9A1.

There are also four differences in sequence between the inter-domain linker of HsALDH9A1 and GmALDH9A2 (Figure 5) but none of them is related to residues establishing H-bonds to βE strand or to residues from catalytic and coenzyme domains. This inter-domain linker serves as a hook in the formation and stabilization of the coenzyme binding site by interacting with the region αβE as well as a loop within the active site interacting with the aldehyde substrate. In our three HsALDH9A1 structures, the inter-domain linker adopts a unique fold, never observed so far, preventing the binding of any substrate. A drastic rearrangement up to 30 Å is therefore required to access the substrate channel. In order to check whether the observed position of the inter-domain linker was independent of the crystallization conditions at acidic pH, we measured the enzyme activity in sodium citrate, pH 5.6. The enzyme still displayed 15% activity and TMABA binding could be measured at this pH by MST (*K*_d_ of 2.6 ± 0.2 mM). Therefore, HsALDH9A1 which was able to crystallize in three different crystals forms, was active. The same fold of the inter-domain linker in the three crystal forms suggests that this linker exhibits such conformation in the apoform. Therefore, a switch corresponding to a rearrangement up to 30 Å must occur for substrate binding followed by NAD^+^ binding. A fine control mechanism of the enzyme may exist as previously reported for ALDH5 [31], in which the substrate channel is blocked by the catalytic loop through a disulfide bond formation between the catalytic cysteine Cys^340^ and the surrounding Cys^342^. However, no similar disulfide bond is formed in HsALDH9A1.

## Materials and methods

### Expression and purification

Human *ALDH9A1* ORF was amplified using gene-specific primers (5′-TTAGGATCCGATGAGCACTGGCACCTTC-3′ and 5′-ACACTCGAGGTCAAAAAGCAGATTCCACA-3′) and Accuprime *Pfx* polymerase (Life Technologies) and further ligated into a pCDFDuet vector (Merck Millipore) using BamHI and XhoI and transformed into T7 express *Escherichia coli* cells (New England Biolabs) and Rosetta2 (DE3) pLysS cells (Merck Millipore). Cells were grown at 37°C in LB medium, at OD_600_ = 0.5 the cultures were supplemented with 0.5 mM isopropyl-β-thiogalactopyranoside for protein expression, and incubated at 20°C overnight. Recombinant ALDHs were purified on HisPur Cobalt or NiNTA Spin Columns (Thermo Fisher Scientific) in 20–50 mM Tris/HCl pH 7.5 with or without 150 mM NaCl. Enzymes were concentrated using Amicon 30 kDa filters (Merck Millipore) and further purified by gel filtration chromatography on a HiLoad 26/60 Superdex 200 column.

### Affinity and thermal stability determination

MST method was used to determine binding affinities for TMABAL, BAL, and NAD^+^. HsALDH9A1 was labeled with the His-Tag Labeling Kit RED-tris-NTA (100 nM dye + 200 nM His-tagged protein) for 30 min. The labeled proteins were adjusted to 50 nM with 50 mM Tris/HCl buffer at pH 7.5 supplemented with 0.05% Tween. A series of sixteen 1:1 ligand dilutions was prepared using the identical buffer. Measurements were done on a Monolith NT.115 instrument (NanoTemper Technologies). Data of three independently pipetted measurements were analyzed.

Thermostability was measured by nano differential scanning fluorimetry on a Prometheus NT.48 instrument (NanoTemper Technologies) with a back-reflection aggregation detection at a range from 20 to 95°C and with a heating rate of 1°C.min^−1^. Protein unfolding was followed by tryptophan fluorescence intensity at 330 and 350 nm in various buffers covering pH range of 7.0–9.0 in the presence or absence of 100 mM NaCl and 5% (v/v) glycerol. The melting temperature (*T*_m_) was determined by detecting the maximum of the first derivative of the fluorescence ratios (F_350_/F_330_) after fitting experimental data with a polynomial function. Data were measured in triplicate. Effect of presence of coenzyme, substrate, or product was measured using Tycho NT.6 instrument with a heating rate of 30°C.min^−1^.

### Enzyme kinetics

Enzyme activity was measured by monitoring the NAD(P)H formation (ε_340_ = 6.22 mM^−1^.cm^−1^) on an Agilent UV-Vis spectrophotometer 8453 (Agilent) at 30°C. Britton–Robinson buffers in the pH range of 6–10 and adjusted to a constant ionic strength of 0.15 M were used to determine pH optimum.

Aliphatic and aromatic aldehydes, BAL chloride together with APAL and ABAL diethylacetals were purchased from Sigma–Aldrich. Diethylacetals of GBAL, 3-guanidinopropionaldehyde (GPAL), 3-(trimethylamino)propionaldehyde (TMAPAL), and 4-(trimethylamino)butyraldehyde (TMABAL) were synthetic preparations [11,32]. Free aminoaldehydes were prepared by heating their acetals in a plugged test tube with 0.2 M HCl for 10 min [33]. Substrate screening was done upon addition of various aldehydes at a final concentration of 1 mM in 150 mM sodium pyrophosphate, pH 7.5 and 1 mM NAD^+^. Saturation curves for substrates were measured using 1 mM NAD^+^. Kinetic constants were determined using GraphPad Prism 5.0 data analysis software (www.graphpad.com) by fitting data to the Michaelis–Menten equation. When substrate inhibition was observed, data were analyzed by nonlinear regression using Michaelis–Menten equation that accounts for partial substrate inhibition: v = *V*_max_.[S]/(*K*_m_+ [S].(1+[S]/*K*_i_)), where v is the determined initial velocity, *V*_max_ is the maximal velocity, [S] is the concentration of the substrate, *K*_m_ is the substrate concentration at half-maximal velocity, *K*_i_ is the substrate inhibition constant. Saturation curve for NAD^+^ was measured using 0.1 mM TMABAL, which is a sub-saturating concentration providing the maximal experimentally attainable activity and is not affected by a substrate inhibition. Therefore, the kinetic constants calculated for the coenzyme are only apparent.

### Crystallization and structure determination

Crystallization conditions were screened using Qiagen kits (Valencia) using HsALDH9A1 purified at 28 mg.ml^−1^ in 50 mM Tris/HCl, pH 7.5 and 150 mM NaCl. Crystals (P2_1_2_1_2 space group) were obtained in hanging drops by mixing equal volumes of protein solution containing 50 mM NAD^+^ and a precipitant solution containing 12% (*w/v*) PEG 4000, 0.1 M sodium citrate pH 5.6, and 2.5% isopropanol. The two other crystal forms (P2_1_ and C2 space groups) were obtained by mixing equal volumes of protein solution containing 10 mM TMABA and 50 mM NAD^+^ and a precipitant solution of 14% PEG 4000 in 0.1 M sodium citrate pH 5.6. The cryoprotectant solution comprised 22% PEG 400 in addition to the precipitant solution. Crystals were flash-frozen in liquid nitrogen and diffraction data were collected at 100 K on the Proxima 1 beamline at SOLEIL synchrotron (Saint-Aubin, France). Diffraction intensities were integrated with the program XDS [34] and data quality was assessed using the correlation coefficient *CC*_1/2_ [35]. The crystal structures were determined by performing molecular replacement with Phaser [36], using the dimer of cod liver ALDH9 (PDB ID: 1A4S) as a search model [19]. Model was refined with NCS restraints and TLS using Buster 2.10 [37]. Electron density maps were evaluated using COOT [38].

### Accession numbers

The atomic coordinates and structure factors of HsALDH9A1 have been deposited in the Protein Data Bank under accession code 6QAK for the P2_1_2_1_2 space group, 6QAO for the P2_1_ space group and 6QAP for the C2 space group.

## Abbreviations

ABAL: 4-aminobutyraldehyde
ABALDH: ABAL dehydrogenase
AHBAL: 4-amino-2-hydroxybutyraldehyde
APAL: 3-aminopropionaldehyde
BAL: betaine aldehyde
DOPAL: 3,4-dihydroxyphenylacetaldehyde
E.C.: Enzyme Commission
GABA: 4-aminobutyric acid
GBAL: 4-guanidinobutyraldehyde
GHBAL: 4-guanidino-2-hydroxybutyraldehyde
MST: microscale thermophoresis
NAD^+^: β-nicotinamide adenine dinucleotide
nanoDSF: nano-differential scanning fluorimetry
NCS: Non-Crystallographic Symmetry
PDB: Protein Data Bank
PPARα: peroxisome proliferator-activated receptor α
TLS: Translation/Liberation/Screw
TMABA: γ-trimethylaminobutyric acid
TMABAL: γ-trimethylaminobutyraldehyde
TMABALDH: TMABAL dehydrogenase

## References

[1] Sophos N.A., Pappa A., Ziegler T.L. and Vasiliou V. (2001) Aldehyde dehydrogenase gene superfamily: the 2000 update. Chem. Biol. Interact. 130–132, 323–337 10.1016/S0009-2797(00)00275-1 11306055

[2] Brocker C., Vasiliou M., Carpenter S., Carpenter C., Zhang Y., Wang X. (2013) Aldehyde dehydrogenase (ALDH) superfamily in plants: gene nomenclature and comparative genomics. Planta 237, 189–210 10.1007/s00425-012-1749-0 23007552 PMC3536936

[3] Kurys G., Ambroziak W. and Pietruszko R. (1989) Human aldehyde dehydrogenase. Purification and characterization of a third isozyme with low *K* _m_ for gamma-aminobutyraldehyde. J. Biol. Chem. 264, 4715–4721 2925663

[4] Suzuki O. and Matsumoto T. (1987) Purification and properties of diamine oxidase from human kidney. Biogenic. Amines. 4, 237–245

[5] Lin S.W., Chen J.C., Hsu L.C., Hsieh C.L. and Yoshida A. (1996) Human gamma-aminobutyraldehyde dehydrogenase (ALDH9): cDNA sequence, genomic organization, polymorphism, chromosomal localization, and tissue expression. Genomics 34, 376–380 10.1006/geno.1996.0300 8786138

[6] Kikonyogo A. and Pietruszko R. (1996) Aldehyde dehydrogenase from adult human brain that dehydrogenates gamma-aminobutyraldehyde: purification, characterization, cloning and distribution. Biochem. J. 316, 317–324 10.1042/bj3160317 8645224 PMC1217341

[7] Ambroziak W. and Pietruszko R. (1991) Human aldehyde dehydrogenase. Activity with aldehyde metabolites of monoamines, diamines, and polyamines. J. Biol. Chem. 266, 13011–8 2071588

[8] Chern M.K. and Pietruszko R. (1995) Human aldehyde dehydrogenase E3 isozyme is a betaine aldehyde dehydrogenase. Biochem. Biophys. Res. Commun. 213, 561–568 10.1006/bbrc.1995.2168 7646513

[9] McNeil S.D., Nuccio M.L. and Hanson A.D. (1999) Betaines and related osmoprotectants. Targets for metabolic engineering of stress resistance. Plant Physiol. 120, 945–950 10444077 10.1104/pp.120.4.945 PMC1539222

[10] Hulse J.D. and Henderson L.M. (1980) Carnitine biosynthesis. Purification of 4-N′-trimethylaminobutyraldehyde dehydrogenase from beef liver. J. Biol. Chem. 255, 1146–1151 7356654

[11] Vaz F.M., Fouchier S.W., Ofman R., Sommer M. and Wanders R.J. (2000) Molecular and biochemical characterization of rat γ-trimethylaminobutyraldehyde dehydrogenase and evidence for the involvement of human aldehyde dehydrogenase 9 in carnitine biosynthesis. J. Biol. Chem. 275, 7390–7394 10.1074/jbc.275.10.7390 10702312

[12] Jakobs B.S. and Wanders R.J. (1995) Fatty acid beta-oxidation in peroxisomes and mitochondria: the first, unequivocal evidence for the involvement of carnitine in shuttling propionyl-CoA from peroxisomes to mitochondria. Biochem. Biophys. Res. Commun. 213, 1035–1041 10.1006/bbrc.1995.2232 7654220

[13] Mandard S., Müller M. and Kersten S. (2004) Peroxisome proliferator-activated receptor alpha target genes. Cell Mol. Life Sci. 61, 393–416 10.1007/s00018-003-3216-3 14999402 PMC11138883

[14] Wen G., Ringseis R., Rauer C. and Eder K. (2012) The mouse gene encoding the carnitine biosynthetic enzyme 4-N-trimethylaminobutyraldehyde dehydrogenase is regulated by peroxisome proliferator-activated receptor α. Biochim. Biophys. Acta 1819, 357–365 10.1016/j.bbagrm.2012.01.004 22285688

[15] Matsunaga A., Harita Y., Shibagaki Y., Shimizu N., Shibuya K., Ono H. (2015) Identification of 4-trimethylaminobutyraldehyde Dehydrogenase (TMABA-DH) as a candidate serum autoantibody target for Kawasaki disease. PLoS ONE 10, e0128189 10.1371/journal.pone.0128189 26010099 PMC4444320

[16] Sato W., Horie Y., Kataoka E., Ohshima S., Dohmen T., Iizuka M. (2006) Hepatic gene expression in hepatocyte-specific Pten deficient mice showing steatohepatitis without ethanol challenge. Hepatol. Res. 34, 256–265 10.1016/j.hepres.2006.01.003 16490391

[17] Inada T., Koga M., Ishiguro H., Horiuchi Y., Syu A., Yoshio T. (2008) Pathway-based association analysis of genome-wide screening data suggest that genes associated with the gamma-aminobutyric acid receptor signaling pathway are involved in neuroleptic-induced, treatment-resistant tardive dyskinesia. Pharmacogenet. Genomics 18, 317–323 10.1097/FPC.0b013e3282f70492 18334916

[18] Chen V.B., Arendall W.B. III, Headd J.J., Keedy D.A., Immormino R.M., Kapral G.J. (2010) MolProbity: all-atom structure validation for macromolecular crystallography. Acta Crystallogr. D Biol. Crystallogr. 66, 12–21 10.1107/S0907444909042073 20057044 PMC2803126

[19] Johansson K., El-Ahmad M., Ramaswamy S., Hjelmqvist L., Jörnvall H. and Eklund H. (1998) Structure of betaine aldehyde dehydrogenase at 2.1 Å resolution. Protein Sci. 7, 2106–2117 10.1002/pro.5560071007 9792097 PMC2143847

[20] Tylichová M., Kopečný D., Moréra S., Briozzo P., Lenobel R., Snégaroff J. (2010) Structural and functional characterization of plant aminoaldehyde dehydrogenase from *Pisum sativum* with a broad specificity for natural and synthetic aminoaldehydes. J. Mol. Biol. 396, 870–882 10.1016/j.jmb.2009.12.015 20026072

[21] Kopečný D., Končitíková R., Tylichová M., Vigouroux A., Moskalíková H., Soural M. (2013) Plant ALDH10 family: identifying critical residues for substrate specificity and trapping a thiohemiacetal intermediate. J. Biol. Chem. 288, 9491–9507 10.1074/jbc.M112.443952 23408433 PMC3611018

[22] Li W., Yuan X.M., Ivanova S., Tracey K.J., Eaton J.W. and Brunk U.T. (2003) 3-Aminopropanal, formed during cerebral ischaemia, is a potent lysosomotropic neurotoxin. Biochem. J. 371, 429–436 10.1042/bj20021520 12513695 PMC1223282

[23] Murray-Stewart T., Wang Y., Devereux W. and Casero R.A. Jr (2002) Cloning and characterization of multiple human polyamine oxidase splice variants that code for isoenzymes with different biochemical characteristics. Biochem. J. 368, 673–677 10.1042/bj20021587 12398765 PMC1223052

[24] Vujcic S., Diegelman P., Bacchi C.J., Kramer D.L. and Porter C.W. (2002) Identification and characterization of a novel flavin-containing spermine oxidase of mammalian cell origin. Biochem. J. 367, 665–675 10.1042/bj20020720 12141946 PMC1222929

[25] Yoshida M., Tomitori H., Machi Y., Hagihara M., Higashi K., Goda H. (2009) Acrolein toxicity: Comparison with reactive oxygen species. Biochem. Biophys. Res. Commun. 378, 313–318 10.1016/j.bbrc.2008.11.054 19032949

[26] Holt A. and Baker G.B. (1995) Metabolism of agmatine (clonidine-displacing substance) by diamine oxidase and the possible implications for studies of imidazoline receptors. Prog. Brain Res. 106, 187–197 10.1016/S0079-6123(08)61215-7 8584654

[27] Iyer R.K., Kim H.K., Tsoa R.W., Grody W.W. and Cederbaum S.D. (2002) Cloning and characterization of human agmatinase. Mol. Genet. Metab. 75, 209–218 10.1006/mgme.2001.3277 11914032

[28] Zhou Y., Holmseth S., Hua R., Lehre A.C., Olofsson A.M., Poblete-Naredo I. (2012) The betaine-GABA transporter (BGT1, slc6a12) is predominantly expressed in the liver and at lower levels in the kidneys and at the brain surface. Am. J. Physiol. Renal Physiol. 302, F316–F328 10.1152/ajprenal.00464.2011 22071246

[29] Ueland P.M., Holm P.I. and Hustad S. (2005) Betaine: a key modulator of one-carbon metabolism and homocysteine status. Clin. Chem. Lab. Med. 43, 1069–1075 10.1515/CCLM.2005.187 16197300

[30] Hjelmqvist L., Norin A., El-Ahmad M., Griffiths W. and Jörnvall H. (2003) Distinct but parallel evolutionary patterns between alcohol and aldehyde dehydrogenases: addition of fish/human betaine aldehyde dehydrogenase divergence. Cell Mol. Life Sci. 60, 2009–2016 10.1007/s00018-003-3287-1 14523561 PMC11478013

[31] Kim Y.G., Lee S., Kwon O.S., Park S.Y., Lee S.J., Park B.J. (2009) Redox-switch modulation of human SSADH by dynamic catalytic loop. EMBO J. 28, 959–968 10.1038/emboj.2009.40 19300440 PMC2670868

[32] Šebela M., Brauner F., Radová A., Jacobsen S., Havliš J., Galuszka P. (2000) Characterisation of a homogeneous plant aminoaldehyde dehydrogenase. Biochim. Biophys. Acta 1480, 329–341 10.1016/S0167-4838(00)00086-8 11004571

[33] Trossat C., Rathinasabapathi B. and Hanson A.D. (1997) Transgenically expressed betaine aldehyde dehydrogenase efficiently catalyzes oxidation of dimethylsulfoniopropionaldehyde and -aminoaldehydes. Plant Physiol. 113, 1457–1461 10.1104/pp.113.4.1457 12223684 PMC158270

[34] Kabsch W. (2010) XDS. Acta Crystallogr. D Biol. Crystallogr. 66, 125–132 10.1107/S0907444909047337 20124692 PMC2815665

[35] Karplus P.A. and Diederichs K. (2012) Linking crystallographic model and data quality. Science 336, 1030–1033 10.1126/science.1218231 22628654 PMC3457925

[36] Storoni L.C., McCoy A.J. and Read R.J. (2004) Likelihood-enhanced fast rotation functions. Acta Crystallogr. D Biol. Crystallogr. 60, 432–438 10.1107/S0907444903028956 14993666

[37] Bricogne G., Blanc E., Brandl M., Flensburg C., Keller P., Paciorek W. (2017) BUSTER version 2.10.3, Global Phasing Ltd, Cambridge, United Kingdom

[38] Emsley P. and Cowtan K. (2004) Coot: model-building tools for molecular graphics. Acta Crystallogr. D Biol. Crystallogr. 60, 2126–2132 10.1107/S0907444904019158 15572765

